# Repair of UV-induced thymine dimers is compromised in cells expressing the E6 protein from human papillomaviruses types 5 and 18

**DOI:** 10.1038/sj.bjc.6601829

**Published:** 2004-04-27

**Authors:** S Giampieri, A Storey

**Affiliations:** 1 Skin Tumour Laboratory, Cancer Research UK, Centre for Cutaneous Research, 2 Newark Street, London E1 2AT, UK

**Keywords:** UV, HPV, DNA damage repair, p53

## Abstract

Ultraviolet (UV) irradiation is a major mutagenic environmental agent, causing the appearance of DNA adducts that, if unrepaired, may give rise to mutations. Ultraviolet radiation has been indicated as a major risk factor in the development of nonmelanoma skin cancers; however, recent reports have suggested that infections with human papillomaviruses, a widespread family of epitheliotropic DNA viruses, may also contribute to the tumorigeneic process. Here, we investigated whether expression of the E6 protein from different HPV types interfere with the repair of thymine dimers caused by UV-B radiation. Results show that unrepaired DNA damage can be observed in UV-B-irradiated cells expressing the E6 protein of HPV types found in cervical and epithelial cancers. Moreover, such cells have the ability to overcome the G_1_ cell cycle checkpoint induced as a result of unrepaired DNA.

On a worldwide basis, nonmelanoma skin cancers (NMSCs) are the most commonly diagnosed cancers amongst Caucasians (de Villiers *et al*, 1999; Kiviat, 1999). The major contributing factor to the development of NMSCs at sun-exposed sites is through DNA damage caused by ultraviolet radiation (UVR). The importance of effective DNA damage repair is highlighted by studies on patients affected with the inherited disease Xeroderma pigmentosum (XP), who are at greatly increased risk of developing NMSCs at sun-exposed body sites (Ellis, 1997). This propensity is due to disabling mutations in the XP genes, which are involved in the repair of DNA damage via the nucleotide excision repair (NER) pathway. A similar susceptibility to develop NMSCs is observed in patients with the rare genetic disease epidermodysplasia verruciformis (EV) (Orth, 1986), which predisposes to infection with a particular class of HPV (EV-HPV). In EV-patients, skin tumours are found at sun-exposed sites and appear to associate with and arise from HPV-infected lesions. This suggests that EV-HPV types, principally types 5 and 8, may have a role in promoting the tumorigenic process. Likewise, renal transplant recipients (RTRs), who also are subject to extensive HPV-verrucosis, display an increased risk of developing NMSCs at sun-exposed sites (Surentheran *et al*, 1998; Harwood *et al*, 2000), with the tumours arising from wart sites. Overall, these clinical data suggest that, in addition to UV-radiation, HPV infections may contribute towards the appearance of NMSCs at sun-exposed sites. Recently, it has been observed that cells expressing the HPV E6 protein of type 16, which can promote the degradation of the tumour suppressor p53, also display reduced ability to repair DNA damage (El-Mahdy *et al*, 2000). As the repair of UV damage is, at least in part, dependent on the p53 status of the cells (Ford and Hanawalt, 1995, 1997), the failure to repair DNA damage effectively may be due to the cells being functionally p53 null.

The ability to promote degradation of p53 is however restricted to a handful of HPV types such as 16, 18 and 33, which are commonly associated with the development of cervical cancers. Conversely, other HPV types, more commonly associated with NMSCs development, do not possess this activity (Elbel *et al*, 1997; Jackson and Storey, 2000). It was therefore of interest to determine whether the repair of DNA damage was compromised in cells expressing the E6 protein of other HPV types which do not promote p53 degradation, but are yet associated with the development of NMSCs at sun-exposed sites. To this aim, we investigated the repair of UV-B-induced thymine dimers in wild-type p53 cells that express the E6 proteins from a variety of EV and other cutaneous HPV types. These included HPV type 5, associated with NMSCs of EV-patients; type 10, found in plantar warts; type 18 that is associated cervical cancers and type 77, found in NMSCs of RTRs. Our results show that cells expressing HPV 5 and 18 E6, but not HPV 10 or 77 E6, display a reduced ability to repair thymine dimers, compared to the control cells. Furthermore, cells expressing HPV 5 or 18 E6 are able to bypass the UV-induced G_1_/S checkpoint despite the presence of unrepaired thymine dimers, which may ultimately result in incorrect replication of the genetic material. We extended these initial findings to show that this impairment of thymine dimers repair was restricted to cancer-associated HPVs, and did not occur in cells expressing the E6 protein from EV-HPV types such as types 23, 24 and 49 that are rarely found in cancers.

## MATERIALS AND METHODS

### Plasmids and cloning

The coding sequences of the E6 genes of HPV type 5, 10, 18 and 77 were excised from pcDNA 3 vectors described previously (Jackson *et al*, 2000). The fragments obtained were purified and subcloned into the *Bam*HI–*Eco*RI sites of the bicistronic vector pIres (Clontech, Oxford, UK). The E6 genes of HPV 23, 24 and 49 were amplified from pBR322 (types 23 and 24) and pGem 4 (type 49) plasmids by PCR using the following primers:


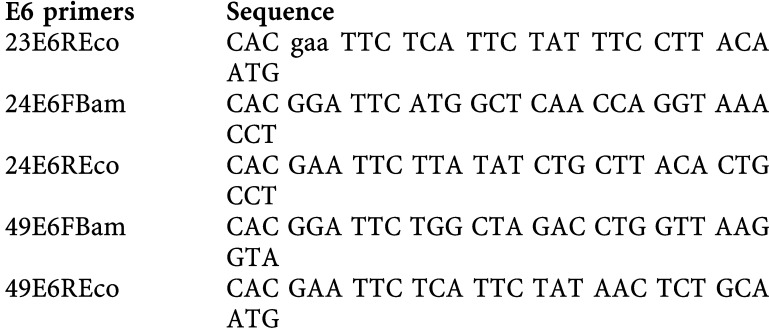


The products were then digested with *Bam*HI and *Eco*RI enzymes, purified and inserted into the *Bam*HI–*Eco*RI sites of the pIres-neo vector (Invitrogen, Paisley, UK).

### Cell culture

Cells lines expressing the control plasmid pIres or its derivatives containing the E6 coding sequence of HPV 5, 10, 18, 77, 23, 24 and 49 were obtained by transfecting HT1080 cells that have wild-type p53 (Rasheed *et al*, 1974) by calcium phosphate method (Wigler *et al*, 1979). Cells were grown at 37°C in 10% CO_2_/90% air in DMEM (Gibco BRL, Paisley, UK) supplemented with 10% foetal calf serum. Selection of transfected cells was carried out for 2 weeks by addition of G418 to the culture media at a final concentration of 350 *μ*g ml^−1^.

### Treatment with UV irradiation

Prior to irradiation, DMEM was removed by washing the cells twice with PBS, which was then completely removed. A CL-1000 M ultraviolet crosslinker (UVP) with 302/310 nm UVB lamps was used to irradiate cells with a single dose of 10 mJ cm^−2^. After irradiation, fresh medium was added to the cells, which were cultured as described until they were harvested.

### Immunoassay for repair of thymine dimers

Thymine dimers present in cellular DNA were detected and quantified using the following standard protocol.

From each cells line, 5 × 10^5^ cells were plated and 16 h later either UV-B irradiated at 10 mJ cm^−2^ or left unirradiated. Total genomic DNA was extracted by using the Nucleon BACC DNA purification kit, according to the manufacturers' instructions. Slot blots of different amounts of human DNA (100–1000 ng) were hybridised to demonstrate a linear relationship between DNA on the filter and signal. To ensure complete denaturation of the DNA, 5 M NaOH was added to the DNA solution to reach the final concentration of 0.4 M NaOH. All samples were then brought to the final volume of 50 *μ*l by addition of 0.4 M NaOH and then the mixture was incubated at 80°C for 30 min. DNA was transferred to a nitrocellulose filter (Hybond XL) using a vacuum blotter (Bio-Dot microfiltration apparatus, Bio-Rad, Hemel Hempstead, UK).

The membrane was allowed to air-dry for 1 h at room temperature, then neutralised for 5 min in 2 × SSC. The filter was baked at 80°C for 150 min to fix the DNA onto the membrane and then stored at 4°C in TBS-T (10 mM Tris-HCl pH8, 0.1% Tween-20, 200 mM NaCl).

The filter was blocked in 10% milk/TBS-T for 1 h at room temperature, before incubating with mouse monoclonal antibody raised against thymine dimers (MC-062, Kamiya Biomedical, Seattle, WA, USA) diluited 1 : 500 in 5% nonfat milk/TBS-T for 1 h. Following washing, membranes were incubated with a secondary rabbit polyclonal anti-mouse fluorescein (FITC)-conjugated antibody (Dako, Ely, Cambs, UK), diluted 1 : 600 in 5% nonfat milk/TBS-T for 1 h. The filter was then incubated with tertiary swine polyclonal anti-rabbit alkaline phosphatase (AP) conjugated antibody, diluted 1 : 1500 in 5% nonfat milk/TBS-T for 1 h. After washing three times in TBS-T, the signal was detected by overlaying the ECF substrate (APBiotech, Bucks, UK) on the membrane for 7 min, according to the manufacturers' instructions.

To evaluate antibody binding, the filter underwent scanning using the STORM 840 chemofluorescent imaging system. The spots, corresponding to the signal generated from DNA damage, were quantified using the IMAGEQuant programme.

To evaluate uniformity of DNA sample loading, each filter was also hybridised with ^32^P-labeled human *β-*actin. *β-*Actin (25 ng) DNA was radioactively labelled by using the Megaprime kit (Amersham, Bucks, UK), according to the manufacturers' instructions. Prehybridisation was carried out for 4 h at 60°C in 5 × Denhart's solution, 2 × SSC, 1% SDS and 100 *μ*g ml^−1^ of preboiled salmon sperm DNA.

Hybridisation took place for 16 h at 60°C in the same solution to which the radioactive probe is added. Finally, the membrane was washed in 2 × SSC, 1% SDS for 10 min. To quantitate the amount of DNA transferred, the membrane was analysed using the Storm 840 phosphorimaging system (Molecular Dynamics, Bucks, UK). The intensity of the signal was converted to numerical data using the ImageQuant programme. Each experiment was repeated three times to ensure reproducibility and representative areas of the filters were used in each case to measure background fluoresence. From pilot experiments it was established that 200 ng of total genomic DNA reproducibly gave a strong signal well within the linear range of detection. Hence, 200 ng of total genomic DNA were used in all the immunoassays thereafter, following the standard protocol.

### Quantification of DNA damage

The amount of residual DNA damage was assessed by the immunoreactivity with the anti-thymine dimers antibody. The strength of the signal was corrected for the amount of total DNA spotted, as measured by radioactive probe hybridisation. Finally, the value of 100% was arbitrarily assigned to the DNA damage present at 1 s after irradiation, and all the other values were assigned accordingly.

### Immunocytochemistry

Antibodies used for the immunocytochemistry experiments included a mouse monoclonal anti-thymine dimer (Kamiya Biochemicals, Japan), 1 : 100 and rabbit polyclonal anti-cyclin A (Santa Cruz, CA, USA), 1 : 300 as primary antibodies, a goat anti-mouse FITC conjugated (Dako), 1 : 100 and goat anti-rabbit Alexa 546 conjugated (Molecular Probes, Cambridge, UK), 1 : 100 as secondary antibodies.

Cells were plated onto glass coverslips, allowed to attach overnight and treated as required. Cells were fixed by immersion in 4% paraformaldehyde in PBS for 20 min at room temperature, then washed twice with fresh PBS and stored at 4°C in PBS. PBS was removed from the cells and a solution of 0.1% Triton X-100 in PBS overlayed for 5 min at room temperature to allow permeabilisation of the cell membranes. Cells were then washed twice with PBS to remove any residual detergent. To avoid nonspecific reaction with the secondary antibody, cells were incubated with 3% goat serum in PBS for 15 min at room temperature. After removal of the serum in PBS, cells were incubated with primary antibody diluted in PBS for 1 h at 37°C. Cells were then washed three times with PBS and incubated with the secondary antibody diluted in PBS for 1 h at room temperature. Finally, cells were washed three times in PBS, once in water, mounted onto glass slide using Immu-mount mounting medium (Thermo-immuno) and visualised by immunofluorecent confocal microscopy, using the upright Zeiss LSM 510 microscope.

## RESULTS

### Thymine dimer repair is compromised in HPV 5- and HPV 18 E6-expressing cells

**Figure 1:**
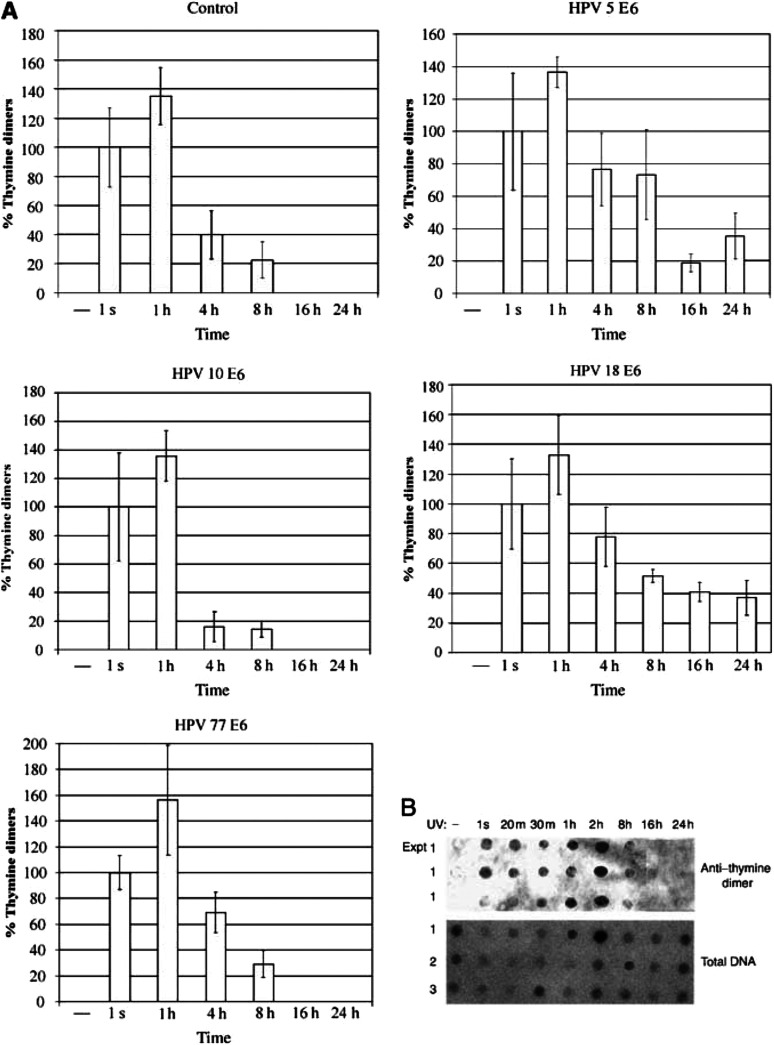
(**A**) Repair of thymine dimers with time in HT1080 cells transfected with the vector control plasmid piers and its derivatives expressing the E6 protein of HPV types 5, 10, 18 and 77. DNA damage repair is compromised in cells expressing HPV 18 E6 or HPV 5 E6, while in cells expressing 10 E6 or 77 E6, the DNA damage repair is comparable to that of the control cell line, HT1080 IRES. The bars represent the standard deviation of three independent experiments. (**B**) Representative dot blots showing the detection of thymine dimers using a specific monoclonal antibody together with quantification of total DNA by Southern blotting. The pIres cells were used in the example shown. The cells were harvested immediately following irradiation (1 s=1 second), at 20 and 30 min or at 1, 2, 16 and 24 h after irradiation, total DNA extracted, spotted onto nitrocellulose membranes and denatured. The membrane was first probed with the anti-thymine dimer antibody, followed by detection using a secondary rabbit polyclonal anti-mouse FITC-conjugated antibody and then incubated with tertiary swine polyclonal anti-rabbit AP. The fluoresence was quantitated using a Storm 840 imaging system and ImageQuant software. Total DNA spotted was determined by Southern blotting.

Cell lines expressing the E6 protein of HPV types 5, 10, 18 and 77 were generated as described in Material and Methods, and expression of the desired gene was verified by RT–PCR (data not shown). In all, 5 × 10^5^ cells from each cell line were UV-B irradiated with 10 mJ cm^−2^ and collected at different time points thereafter. A histogram showing the detection of thymine dimers *vs* time in HT1080 cell lines expressing either the control plasmid or the selected HPV E6-expressing plasmid is shown in Figure 1A

. Representative blots that were used to detect and quantify the thymine dimers, which were then reprobed to determine the total DNA loading, are shown in Figure 1B. In the control cell line, expressing only the neomycin-resistance gene of the backbone vector pIres, the DNA damage signal increased immediately following irradiation, possibly increasing further at around 1 h after stimulus, following a pattern similar to that observed previously (Lu *et al*, 1999). After this time, the signal began to decrease and was undetectable by 16 h postirradiation. This pattern of repair was closely mirrored in cell lines expressing either HPV 10 E6 or HPV 77 E6, in which the level of DNA damage, as judged by the decrease in anti-thymine dimers antibody reactivity, was also undetectable by 16 h post-UV-B treatment. In marked contrast, a different pattern of thymine dimers repair was seen for cell lines expressing either HPV 18 E6 or HPV 5 E6, with between 20 and 30% of the initial signal still detectable 24 h after irradiation (Figure 1A). The delay of DNA damage repair was expected for HPV 18 E6-expressing cell line, as expression of the HPV 18 E6 protein in these cells promotes p53 degradation and the absence of p53 has been already reported to slow DNA damage repair (El-Mahdy *et al*, 2000). However, as demonstrated elsewhere (Jackson and Storey, 2000), HPV 5 E6, in contrast to HPV 18 E6, has been shown to be unable to promote the degradation p53. It cannot be ruled out at this stage that uncharacterised changes in the HT1080 cells may contribute towards this phenotype. These results suggest that HPV 5 E6 could nevertheless impair DNA damage repair through mechanisms that do not involve p53 degradation, which may contribute towards the oncogenic potential of his viral type.

### Thymine dimer repair in cells expressing the E6 protein of different EV-HPV types

HPV 5, in contrast to the other cutaneous HPV tested, belongs to the group of EV-HPV types. It is possible that the oncogenic potential of specific EV types could partly be explained in terms of defective DNA damage repair. To test this possibility, additional cell lines were generated that expressed other EV E6 genes, namely of types 23, 24 and 49, which belong to different clusters within the EV group. Their ability to interfere with the repair thymine dimers was investigated and compared to that of cells expressing the HPV 5 or HPV 18 E6 protein. In addition, for this experiment, the time course was extended to 48 h postirradiation to assess the extent to which DNA damage repair was delayed. In contrast to HPV 5 and 8, which are strongly associated with the development of NMSC in EV patients, types 23, 24 and 49 have been rarely found in the tumours of EV patients, although their presence has been reported in NMSC of RTRs (Harwood *et al*, 2000). It was of interest therefore to establish whether delayed thymine dimers repair by the E6 protein was a property shared between all EV types, or whether only certain types that are frequently associated with cancers, such as HPV 5, possess this activity.

**Figure 2:**
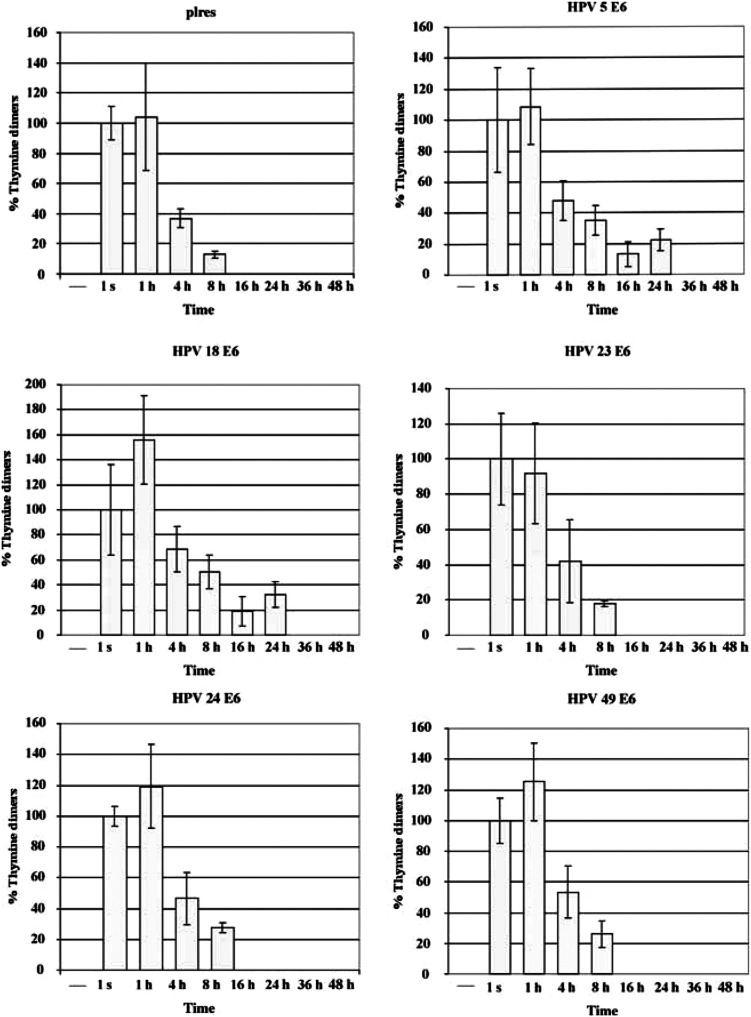
DNA damage levels in HT1080 cell lines expressing diverse EV HPV types. Repair of thymine dimers with time in HT1080 cells transfected with the pIres plasmid or its derivatives expressing the E6 protein of HPV types, 5, 18, 23, 24 and 49. In all cell lines, DNA damage is completely repaired by 36 h. DNA damage repair is delayed in cells expressing HPV 18 E6 or HPV 5 E6, while in cells expressing the E6 protein of EV HPV types 23, 24 or 49, the DNA damage repair rate is comparable to that of the vector control cell line, HT1080 IRES. The bars represent the standard deviation from the mean of three independent experiments.

For this experiment, 5 × 10^5^ cells from each cell line were UV-B irradiated at 10 mJ cm^−2^, the cells were collected at different time points, and the presence of thymine dimers assessed as before. As expected, in the HT1080 IRES control cell line (Figure 2

), the time course curve of the DNA damage signal was in accordance with previous results and repair of thymine dimers was complete by 16 h postirradiation. Likewise, cells expressing HPV 5 E6 and HPV 18 E6 maintained 20–30% of the initial damage at 24 h after irradiation, in good agreement with earlier experiments. Extending the time course from Figure 1A showed that the DNA damage, however, appeared to be mostly if not completely repaired by 36 h in both cells lines, as judged by the decrease in anti-thymine dimer antibody reactivity in our assay system. Cells expressing the HPV 23, 24 and 49 E6 genes displayed a similar pattern of DNA damage repair to that observed in the control cell line, with the repair of thymine dimers being judged to be complete by 16 h postirradiation. These results suggest that the ability to compromise DNA damage repair does not extend to all EV-HPVs, but may be exclusive to types, such as 5, which are more frequently associated with tumour development in EV.

### UV-damaged cells expressing HPV 5 or HPV 18 E6 bypass the G_1_ cell cycle checkpoint

If DNA damage is not repaired correctly, mutagenic events might occur. For these potentially tumour-promoting mutations to have physiological importance in tumour formation, the cells that would normally be growth arrested by UV treatment would have to re-enter the cell cycle in order to replicate the mutated DNA and generate progeny cells with tumorigenic potential. A long-term growth arrest or failure of a damaged cell to divide prevents mutagenic changes introduced during the repair process from being passed on to the progeny cells. Therefore, it was then of interest to determine whether the E6-expressing cells that retained unrepaired DNA damage had resumed cycling.

Under normal conditions, repair of DNA damage would be completed before the onset of the S phase. To investigate whether cells displaying unrepaired thymine dimers were able to enter S phase following UV-induced damage, the expression of the cell cycle progression marker cyclin A in these cells was assessed by immunocytochemical staining. Cyclin A expression is usually repressed during early G_1_ and in quiescent (G_0_) cells, while marked upregulation of the protein is required, starting from late G_1_ phase, for progression to S phase and its expression is maintained through to mid M phase. Transcriptional repression of cyclin A expression results in a G_1_ arrest which can be relieved by ectopic expression of cyclin A (Fajas *et al*, 2001) and induction of G_1_ arrest correlates with reduced expression of cyclin A (Decker *et al*, 2003).

**Figure 3:**
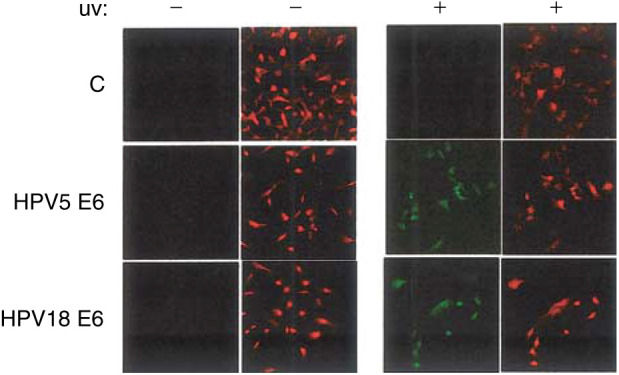
Colocalisation of Cyclin A and thymine dimers in UV-irradiated cells expressing HPV 5 and 18 E6. When cells were released from cell cycle arrest by the addition of medium containing serum, unirradiated cells transfected with empty vector plasmid (C); HPV 5 E6 or HPV 18 E6 did not display presence of thymine dimers. Cyclin A was expressed in many, but not all, cells from each cell line, consistent with recovery from a growth-arrested phenotype. After UV irradiation, cells harbouring the vector plasmid do not display thymine dimers presence and have re-entered the cell cycle, as indicated by cyclin A staining. Cells expressing either the HPV 5 E6 or HPV 18 E6 genes have also resumed cycling, as indicated by the cyclin A staining. However, many of these cyclin A-expressing cells are also immunopositive for thymine dimers, suggesting that these cells have re-entered the cell cycle despite carrying unrepaired DNA damage.

To determine whether cyclin A was still expressed in UV-irradiated cells harbouring thymine dimers, 10^4^ cells from the piers, pIres∷5E6 and pIres∷18E6 lines, were seeded onto glass coverslips and allowed to attach overnight. The following day, the cells were serum starved for 3 days in order to arrest the cells in G_0_, when expression of cyclin A is no longer observed. For each cell line, one coverslip was fixed at that time to verify the cell cycle arrest and lack of cyclin A expression (not shown). Cells on the other coverslips were released from cell cycle arrest by addition of fresh serum-containing media, or irradiated at 10 mJ cm^−2^ and then cultured in fresh serum-containing media for 24 h and then fixed for further analysis. Cyclin A expression and thymine dimers presence were then analysed by fluorescent immunocytochemistry. As expected, no thymine dimer expression could be observed in any of the unirradiated cells, irrespective of the cell line tested (Figure 3

). In all cell lines, nonirradiated cells released from cell cycle blockage by addition of serum-containing medium showed a readily detectable cyclin A within the nuclear compartment (Figure 3). In UV-irradiated HT1080 pIres cells cultured for 24 h in the presence of serum, strong nuclear staining for cyclin A was observed in most cells, while others only showed a weak diffuse staining. This pattern is consistent with recovery from G_1_ growth arrest, which takes place after UV irradiation. None of the UV-irradiated HT1080 IRES cells were positive for thymine dimers. This is in agreement with previous findings that showed, in this cell line, that these adducts were no longer detectable at 16 h after UV irradiation and indicates that these cells did not enter late G_1_/S phase while containing detectable levels of thymine dimers (Figure 3).

In marked contrast in HPV 5- or HPV 18 E6-expressing cells, a proportion of the population showed strong nuclear staining for cyclin A, suggesting that cycling had resumed in these cells. Within these cyclin A-positive cells, only a few showed absence of nuclear staining for thymine dimers, suggesting that in such cells the DNA damage had been completely repaired. However, in the majority of these cells that were cyclin A positive, significant thymine dimers nuclear staining could be readily observed, indicating that these cells are able to bypass cell cycle checkpoints despite the presence of unrepaired thymine dimers (Figure 3).

## DISCUSSION

This study has revealed that impairment of thymine dimers repair after UV irradiation was observed in cells expressing the E6 protein of HPV types 5 and 18. This suggested that these HPV types may participate in the tumour development not only by interfering with UV-induced apoptosis (Jackson *et al*, 2000; Jackson and Storey, 2000) but could also contribute to this process by hindering the DNA damage repair process. Furthermore, interference with the cell cycle responses is observed in both HPV 5- and HPV 18 E6-expressing cells. While these findings could be partly explained by the inactivation of p53 functions in cells expressing HPV 18 E6, our results reveal that this constitutes a novel activity for HPV 5 E6. Interestingly, as HPV 5 E6 does not promote p53 degradation, expression of this protein nevertheless appears sufficient to bypass the cell cycle arrest response enforced by p53 via p21 expression (Jackson and Storey, 2000), without causing degradation of either protein. This suggests that HPV 5 E6 protein might influence other molecules involved in the regulation of cell cycle progression. Our observation on cyclin A immunoreactivity, showing that cells expressing such viral oncogenes were able to bypass the cell cycle checkpoints after UV irradiation while still retaining unrepaired DNA damage, suggested that these findings could be of physiological relevance. In nonarrested cells, the unrepaired DNA could potentially be used as a template for DNA polymerases, such as DNA pol *ξ* and Pol *η*, that lack or have reduced proofreading capabilities (Lehmann, 2000). This may ultimately result in incorrect genetic information being passed to the daughter cells, possibly translating in an increased rate of mutagenesis and genomic instability. Indeed UV radiation might cause mutations in specific genes involved in processes such as apoptosis and cell proliferation, thus causing their deregulation or inactivation. Indeed, p53 gene is the main target of UV-induced mutations, such as C → T or CC → TT transitions, which are clustered around nine mutation hot spots (Brash *et al*, 1991, 1997) and are frequently found in SCC lesions. Similarly, SCC occurring at sun-exposed sites harbour UV-associated mutations at specific positions of the Ha-*ras* oncogene (Pierceall *et al*, 1991). It would be of interest to determine the HPV status in such tumours, as this might be correlated with tumorigenic progression.

In our assays, the quantity of thymine dimers detected was maximal 1 h after irradiation and not when the cells were harvested immediately after being irradiated, as might be expected. This apparent increase has also been observed in other studies (Eller *et al*, 1997; Therrien *et al*, 1999), although how this is brought about is not clear. One possibility is that UV irradiation also generates other adducts such as 6-4 photoproducts that are bulkier than thymine dimers and distort the DNA helix more grossly. If such an adduct occurred in the proximity of a thymine dimer, it is possible that the anti-thymine dimer antibody may no longer be able to recognise the dimer. As the repair of 6-4 photoproducts is faster than that of thymine dimers, it is possible that this leads to the apparent increase in thymine dimers at the 1 h time point. The results of the DNA damage repair time course extended to 48 h postirradiation showed that DNA damage could not be detected in cells expressing HPV 5 and HPV 18 E6 beyond the 24 h time point. The failure to detect thymine dimers beyond 24 h postirradiation may be the result of either the repair process having resumed and being completed between 24 and 36 h. Alternatively, it may be a consequence of ‘diluting’ effect within a growing cell population to a point where the assay lacks sufficient sensitivity to detect the signal generated by the proportionally ever lower number of damage sites per cell.

The ability to inhibit UV-induced apoptosis appears to be a common property shared by diverse HPV E6 proteins, including HPV 5, 10, 18 and 77. This viral activity may represent the baseline upon which addition viral activities that predispose to malignant change can be appended. Our results show that in addition to antiapoptosis, HPV 5 and HPV 18 E6 can also promote a delay in thymine dimers repair. Furthermore, our findings showed that the E6 protein of other cutaneous HPV, such as type 10 and 77, and EV types 23, 24 and 49 did not share this activity. Indeed, previous studies (Proniewska and Jablonska, 1980) also reported that UV-induced DNA repair synthesis was unaffected in patients infected with HPV types 3 or 4. This suggests that such activity was not a common property shared by all HPVs, but rather may be exclusive to certain types, possibly those that are more frequently associated with malignancy. Nevertheless, it is important to note that this constitutes the first report of an EV type-promoting delay in the DNA damage repair process. How the HPV 5 E6 protein can interfere with the repair mechanism remains to be elucidated. As HPV type 5 is frequently associated with the development of squamous cell carcinomas at sun-exposed site in EV patients, it is tempting to speculate that this property could at least in part explain how infections with this EV type may promote the carcinogenic process initiated by UV irradiation. Furthermore, it is possible that both the antiapoptotic and the delayed repair activities could potentially contribute HPV 5 to tumour development.
